# Rapid isolation and cryo‐EM characterization of synaptic vesicles from mammalian brain

**DOI:** 10.1002/2211-5463.13475

**Published:** 2022-09-04

**Authors:** Kang Du, Liqiao Hu, Pei Wang, Yanhong Xue

**Affiliations:** ^1^ Key Laboratory of Molecular Biophysics of the Ministry of Education, College of Life Science and Technology Huazhong University of Science and Technology Wuhan China; ^2^ National Laboratory of Biomacromolecules, CAS Center for Excellence in Biomacromolecules, Institute of Biophysics Chinese Academy of Sciences Beijing China

**Keywords:** cryo‐EM, isolation, mammalian brain, synaptic vesicles

## Abstract

Synaptic vesicles (SVs) store and release neurotransmitters at chemical synapses. Precise regulation of SV trafficking, exocytosis and endocytosis is crucial for neural transmission. Biochemical characterization of SVs, which is essential for research into neurotransmitter uptake and release, requires effective *in vitro* isolation methods. Here, we describe an improved and simple purification protocol for isolating SVs from mouse brain within 6 h, achieving a yield of approximately 0.4 mg of SVs per single brain. The use of track‐etch membrane filtration and iodixanol cushion ensured the uniform morphology of SVs and low contaminants in the sample. Cryo‐electron microscopy was used to show that the *in vitro* isolated SVs retained intact membrane‐associated proteins, and observation of SVs in hippocampal neurons using cryo‐electron tomography confirmed the abundance of protein coating. Thus, our protocol allows effective isolation of SVs from small volumes of mammalian brain tissue, and the properties of the isolated SVs are close to those *in vivo*, making them suitable for biochemical analysis.

## Graphical abstract

Synaptic vesicles (SVs) are organelles at synaptic terminals that store and release neurotransmitters. Here, we introduced a rapid purification protocol for isolating SVs from mouse brain within 6 h. The isolated SVs were characterized by cryo‐electron microscopy imaging and showed good integrity.

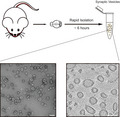

**Abbreviations**
cryo‐EM: cryo‐electron microscopy
cryo‐ET: cryo‐electron tomography
Rab3A: Ras‐related protein Rab‐3A
SEC: size exclusion chromatography
SV2C: synaptic vesicle glycoprotein 2C
SV: synaptic vesicle
vAChT: vesicular acetylcholine transporter
vGAT: vesicular GABA transporter
vGLUT: vesicular glutamate transporter
VPP: Volta phase plate

Synaptic vesicles (SVs) are special organelles at the presynaptic terminal that store and release neurotransmitters. Neurotransmitters encased in SVs are released into the synaptic cleft through SVs exocytosis, where they are recognized by corresponding receptors on the postsynaptic membrane to accomplish the transmission of neural signals [1, 2]. Reinternalization and recycling of SVs follow the exocytosis.

Comprising specific compartments in the mammalian brain, the important role of SVs is structurally and functionally predominated via complicated protein/component and interactions. Neurotransmitter transporters are transmembrane proteins that are distributed on SVs and responsible for diverse substrates [3], such as vesicular glutamate transporters (vGLUT1‐3), vesicular acetylcholine transporter (vAChT) and vesicular GABA transporter (vGAT). Vacuolar‐type H+‐ATPase (v‐ATPase) resides in SVs to maintain an electrochemical gradient. Additionally, various SV‐associated proteins also orchestrate vesicle behavior at the presynaptic side, including trafficking, as well as exo‐ and endocytosis. For example, the SNARE complex is well characterized with respect to facilitating membrane fusion [4]. Recent studies based on SV mass spectrometry provided a complicated composition, and more than 1000 proteins were identified [5]. Although many proteins have been identified, little is known about the membrane structure of SVs, such as the concentration of membrane‐associated proteins and the surface density of important proteins. Thus, *in vitro* purification of SVs is beneficial for biochemical analysis and comprehensive understanding the regulation of SVs.

Synaptic vesicles are spheroidal organelles of 40–50 nm in diameter and are highly abundant in brain tissue. SV‐associated proteins contribute approximately 5% of the total content of the mammalian central nervous system [6]. Current isolation protocols based on multistep centrifugation and column chromatography [6, 7, 8] or beads based immunoaffinity [9, 10] have been reported and directed a successful production of SVs from various tissues or cultured cells. However, the purity and yield are uncompromising. Here, we developed a rapid and simple purification strategy with effective filtration and gentle centrifugation to obtain considerable SVs with high purity. The stable SVs also allowed a suitable cryo‐electron microscopy (cryo‐EM) sample preparation and exhibited an intact, protein‐rich membrane structure. *In situ* morphological observation of SVs via cryo‐electron tomography (cryo‐ET) confirmed that our protocol enables a rapid and high‐quality obtention of SVs from the mammalian resources.

## Materials and methods

All animal experiments were approved by Institutional Animal Ethics Committee of Huazhong University of Science and Technology (License No. SYXK2021‐0057).

### Isolation of synaptic vesicles from mouse brain

Synaptic vesicles were isolated from the mouse brain. Here, we present an improved and simple purification protocol for isolating SVs from mouse brain within 6 h, which finally allows approximately 0.4 mg of SVs per single brain.

The detailed procedures:

1. Remove the brain from one mouse (6–8‐week‐old/C57BL). Strip off the cerebellum and hypothalamus and then place the cerebrum in ice‐cold homogenization buffer. Wash the cerebrum with homogenization buffer [320 mm sucrose, 4 mm Hepes, protease inhibitor (EDTA‐free), pH 7.4, filtered through a 0.22‐μm syringe filter, made fresh on the day]. (In the brain, the cortex is the area with the highest abundance of neurons. Removal of the cerebellum and hypothalamus can reduce contamination in final sample.)
2. Homogenize the cerebrum in 20 mL of ice‐cold homogenization buffer, using a glass homogenizer with 20 strokes at 1200 r.p.m.
3. Centrifuge the sample at 1000 ***g*** for 15 min at 4 °C (Centrifuge 5804R; Eppendorf, Hamburg, Germany). Discard the pellet (P1) and collect the supernatant (S1). (This step is purposed to remove heavy cellular components.)
4. Centrifuge the supernatant (S1) at 15 000 ***g*** for 25 min at 4 °C (Centrifuge 5804R; Eppendorf). Discard the supernatant (S2) and resuspend the pellet (P2) with 1 mL of homogenization buffer. (After centrifugation, synaptosomes are enriched in P2, whereas numerous small cell fragments such as microsomes and cell membrane are released into S2. S2 should be discarded to ensure the purity of the sample.)
5. Place the fraction into glass homogenizer, add 9 mL of ice‐cold Buffer R [7.7 mm Hepes, protease Inhibitor (EDTA‐free), 1 mm MgCl_2_, 1 mm ATP, pH 7.4, filtered through a 0.22‐μm syringe filter, made fresh on the day] and quickly homogenize the fraction for 10 strokes at 900 r.p.m. Transfer the fraction to a 15‐mL tube. Place the tube on ice for 20 min. After that, add 544 μL of 2.5 mol·L^−1^ KCl to the tube. (To release SVs from the synaptosomes, we performed osmotic lysis. Osmotic lysis could break the membranes of synaptosomes with few damages to SVs inside. Osmotic lysis was stopped when KCl buffer was added.)
6. Centrifuge the lysate at 15 000 ***g*** for 5 min at 4 °C (Eppendorf; Centrifuge 5804R) and collect the supernatant. Then, centrifuge the supernatant at 70 000 ***g*** for 30 min at 4 °C (Optima XE 90 with a SW40Ti rotor; Beckman Coulter, Brea, CA, USA), collect the supernatant (S3) and discard the pellet (P3). (Compared to the previously reported protocol [6, 7, 8], in the step purposed to remove cracked synaptosomes, we use a higher centrifugal force. We find that a greater centrifugal force can better remove the synaptosomes and other large membrane particles such as mitochondria.)
7. Filter S3 through 0.45‐ and 0.22‐μm polytetrafluoroethylene membranes. Filter the pass flow through 200 nm track‐etch nuclepore membrane (catalog. no. 111106; Whatman, GE Healthcare, Chicago, IL, USA). The flow rate should be approximately 5 mL·min^−1^ with a manual syringe. (Although most contaminants in the sample had been removed after step 6, a few large particles over 100 nm still existed in S3. We need to remove these constituents to ensure a high purity. Different from the irregular pores on polytetrafluoroethylene membrane, pores on track‐etch nuclepore membrane are standard 200 nm. This arrangement of double filtration makes it more difficult for large contaminants to pass through the membranes.)
8. Transfer 9 mL of filtered sample to a SW40 tube (catalog. no. 344057; Beckman Coulter). Gently add 3 mL of 15% iodixanol buffer (15% iodixanol, 150 mm KCl, 10 mm Hepes, 1 mm MgCl_2_, 1 mm ATP, pH 7.4, filtered through a 0.22‐μm syringe filter, made fresh on the day) to the bottom of the tube using a long syringe needle. Place the SW40 tube inside the centrifuge. Centrifuge the sample at 200 000 ***g*** for 3 h at 4 °C (Optima XE 90 with a SW40Ti rotor; Beckman Coulter). Resuspend the pellet with Buffer A (150 mm KCl, 10 mm Hepes, 1 mm MgCl_2_, 1 mm ATP, pH 7.4, filtered through a 0.22‐μm syringe filter, made fresh on the day). (In classic SV purification schemes, SVs are concentrated by direct centrifugation. After ultracentrifugation, SVs would be enriched at the bottom of the centrifuge tube. The high centrifugal force and extrusion of vesicles against each other would result in the breakage of vesicles. In addition, the pellet would be tightly attached to the bottom of the centrifuge tube, making it very difficult for the pellet be resuspended. Even after resuspension, the dispersion of the sample would become very poor, which was detrimental to subsequent experiments. To solve this problem, we developed a soft‐landing design in which an iodixanol cushion was laid at the bottom of the SW40 tube. The iodixanol cushion has a certain viscosity and can serve as a protecting buffer during centrifugation, reducing the mutual extrusion between SVs, protecting the integrity of SVs and making the pellet easier to be resuspended. In addition, benefiting from the high density of the 15% iodixanol buffer, small proteins in the solution can hardly enter the 15% iodixanol solution, which improves the purity of precipitated SVs.)

### Negative staining

Synaptic vesicles sample was applied to a carbon‐coated grid and stained with 2% uranyl acetate. Imaging was performed using a Tecnai Spirit electron microscope (FEI, Hillsboro, OR, USA) operated at 120 kV.

### Cryo‐EM sample preparation

For cryo‐EM, using a Vitrobot Mark IV (FEI), the SV sample was applied to a glow discharged, Quantifoil R1.2/1.3, 300 mesh grid (Electron Microscopy Sciences, Hatfield, PA, USA), blotted for 4–5 s and rapidly frozen in liquid ethane. Imaging was performed with a Titan Krios electron microscope (FEI) operated at 300 kV.

### Preparation of primary neuronal cultures and cryo‐ET

For EM grid preparation, Quantifoil R2/1 gold EM grids (200 mesh with holey carbon film) were plasma cleaned with O_2_ and H_2_ for 30 s and sterilized with UV light for 20 min. The grids were coated with poly‐d‐lysine (A3890401; Gibco, Waltham, MA, USA) overnight, followed by washing with sterilized ddH_2_O six times and adding HBSS solution (catalog. no. 24020117; Gibco) for incubation.

Hippocampal neurons were removed from P0 mice and were digested with papain for 30 min in a water bath at 37 °C. Next, the tissues were gently washed twice with trituration solution and mechanically triturated 15–20 times with a 1‐mL tip to obtain a single cell suspension (avoiding bubbles) [trituration solution (total 5 mL): Glutamax 0.025 mL, fetal bovine serum 0.5 mL, 10% BSA/HBSS 0.5 mL, DNase 0.05 mL in HBSS]. The sample were centrifuged at 200 ***g*** for 5 min at 4 °C and the supernatant was discarded. Dissociated cells were suspended in 1 mL of plating medium (Dulbecco's modified Eagle's medium with 10% fetal bovine serum, catalog. no. 11995065; Gibco) and applied to the cell counter for counting (trypan blue 1 : 1). Dilute the cell suspension with plating medium and seed onto the coverslip with gold EM grids at a low density of 70–100 K cells per dish. Eight hours after seeding, the plating medium was replaced by the culture medium. The culture medium was NeuroBasal (Gibco) supplemented with 2% B27 (Invitrogen, Carlsbad, CA, USA) plus 0.5 mm Glutamax (Invitrogen). Subsequently, half of the culture medium was replaced with fresh culture medium every 3–4 days. Primary neurons were maintained in incubators at 37 °C in 5% CO_2_ until 14–18 days *in vitro*. Imaging of cryo‐EM was performed with a Titan Krios electron microscope (FEI) operated at 300 kV. Tilt‐series were collected in a dose‐symmetric tilting scheme from −51° to +51° with a step size of 3° using serialem [11]. Tilt‐series were collected at a magnification of 53 000×, corresponding to a pixel size of 2.58 Å. The total dose per tilt series was approximately 60 e^−^·Å^−2^.

### Immunoblotting

Samples were prepared with SDS loading buffer and resolved by 12% SDS/PAGE, then transferred for 1 h to 0.45‐mm poly(vinylidene difluoride) membranes. The membranes were blocked with 5% milk in TBST (i.e. Tris‐buffered saline‐Tween 20), then incubated with antibodies in 3% milk TBST for 1 h at room temperature. Antibodies were used: SV2C (catalog. no. ab33892; Abcam, Cambridge, UK), vGlut1 (catalog. no. ab227805; Abcam) and Rab3A (catalog. no. ab3335; Abcam). Following incubation, membranes were washed three times with TBST and incubated with the corresponding secondary antibodies in 3% milk. After the membranes were washed three times again with TBST, they were visualized using enhanced chemiluminescence.

For the gold nanoparticles immunoassay, V‐ATPase E1 Polyclonal Antibody (catalog. no. PA5‐29899; Thermo Fisher; Waltham, MA, USA) and 10 nm BSA gold tracer (catalog. no. 25486; Electron Microscopy Sciences) were used to mark the samples.

## Results

### Rapid and simple purification of synaptic vesicles

**Fig. 1:**
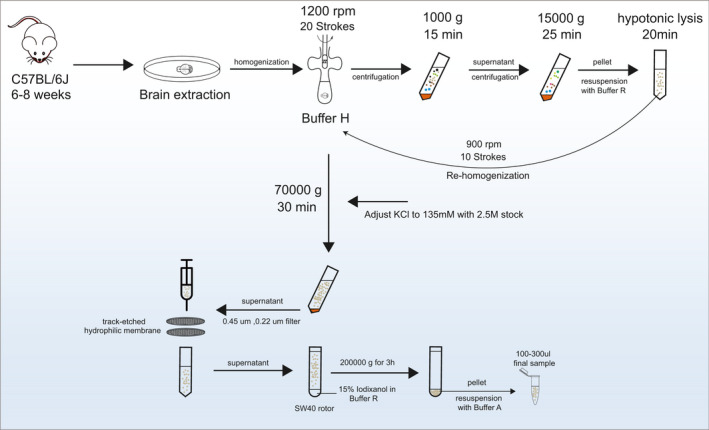
Rapid workflow for isolation of SVs from the mouse brain. Detailed procedures are reported in the Materials and methods.

The isolation of SVs facilitates accurate biochemical characterization and investigations based on *in vitro* experiments, which are essential for understanding neurotransmitter transmission [12, 13]. Several methods have been reported for the direct purification of SVs from the mammalian brain [6, 7, 14]; however, these methods usually require complicated purification processes, and the yield and quality of SVs need to be improved. To this end, we have optimized and established a simple protocol to obtain high‐purity SVs within 6 h or less and without the need for specific instruments (Fig. 1). The protocol allows the extraction of up to 0.4 mg of qualified SVs from a single mouse brain. The obtained SVs exhibited complete morphology and a stable state in the buffer; additionally, efficient removal of organelles or cell debris and proteins/protein complexes provided a suitable sample for electron microscopy observation.

**Fig. 2:**
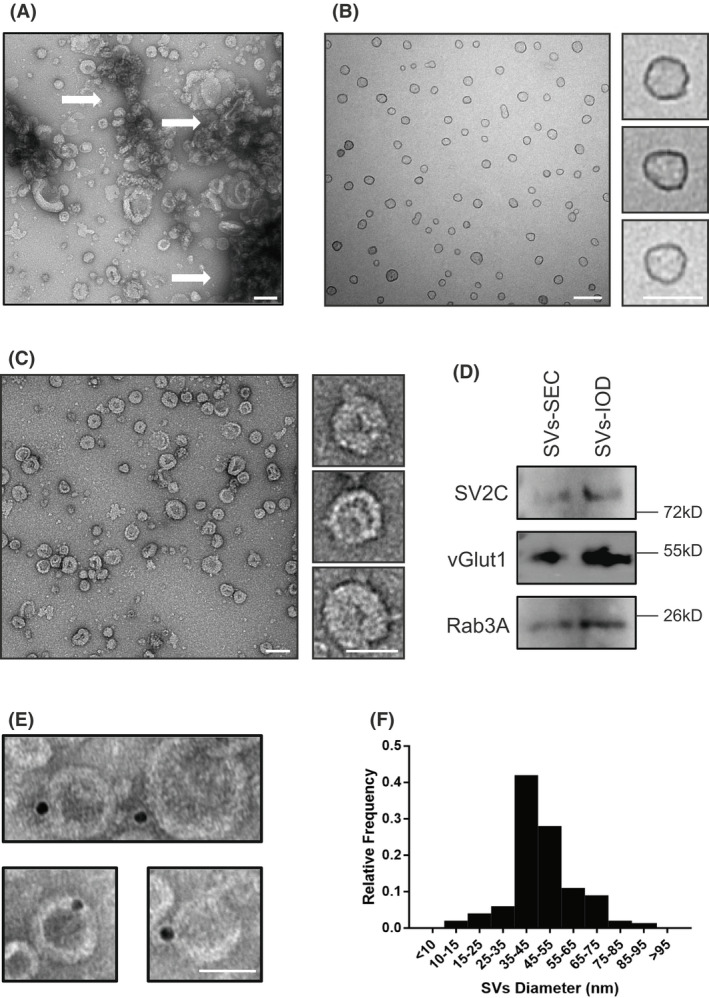
Characterization of *in vitro* isolated SVs. (A) SVs enriched by ultracentrifugation, negatively stained by uranyl acetate. White arrows indicate the aggregation of vesicles. Scale bar = 200 nm. (B) SVs collected through home‐made gel filtration column with Sephacryl S‐1000 beads and negatively stained by uranyl acetate. Scale bar = 200 nm. Zoomed bar = 50 nm. (C) SVs isolated with iodixanol layer, negatively stained by uranyl acetate. Scale bar = 200 nm. Zoomed bar = 50 nm. (D) Protein levels of SV2C, vGlut1 and Rab3A were determined via western blotting. (E) SVs labeled with 10‐nm gold tracer, negatively stained by uranyl acetate. Scale bar = 50 nm. (F) The measurement of the diameter of the purified SVs.

Synaptosomes contain numerous SVs, and suitable hypotonic conditions can promote the final yield. In our protocol, secondary homogenization of synaptosomes after static swelling greatly improved the level of SV release. To effectively remove cell debris and other membrane organelles, the sample was subject to a filter. Our protocol introduces a track‐etch membrane with a completely identical pore size that strictly limits large components, enabling us to easily obtain SVs with a relatively uniform diameter, thus allowing for the omission of density gradient centrifugation. To remove small particles of soluble protein/protein complexes in the sample, SVs were enriched to the bottom of the centrifuge tube by ultracentrifugation. In this procedure, we found at least two problems (Fig. 2A): (a) Although most of the protein contaminants were removed, the collected SVs still contained ribosomal particles, and (b) the pellet enriched by ultracentrifugation caused irreversible aggregation of vesicles and mechanical damage. To further improve the purity of SVs, we introduced a buffer‐layer at the bottom of the centrifuge tube. For this step, 15% iodixanol was determined and glycerol was also the alternative substitute, which achieved the best balance between the yield and the purity of SVs.

Because size exclusion chromatography (SEC) is a widely used strategy for the final purification of SVs in various reported methods [6, 14], we also performed a comparison. We found that, if the starting sample contains large membrane‐structured components, the gel filtration column will be blocked, which raises a higher requirement for the sample to be separated. The column pressure and shearing force of the stationary phase resulted in the elution effect of SV surface proteins (Fig. 2B), which is unfavorable for maintaining the natural shape of SVs and subsequent biochemical analysis.

Based on negative staining, the purified SVs that followed our protocol retained proteins at the membrane; however, vesicles from SEC purification had a smooth surface (Fig. 2B,C). Additionally, the synaptic vesicle protein levels of synaptic vesicle glycoprotein 2C (SV2C), vesicular glutamate transporter 1 (vGlut1) and Ras‐related protein Rab‐3A (Rab3A) from SEC and Iodixanol layer purification indicated marked differences in immunoblotting (Fig. 2D). To further assess the integrity of SVs, we performed a gold‐nanoparticles immunoassay against v‐ATPase and the negative staining results exhibited efficient labeling (Fig. 2E). The measurement of the diameter of purified SVs also showed a major distribution (75%) at 30–50 nm (Fig. 2F).

Taken together, we describe here a simple and effective purification strategy that allows the eligible isolation of SVs from limited animal sources with uncomplicated experimental equipment.

### Characterization of isolated synaptic vesicles via cryo‐EM

**Fig. 3:**
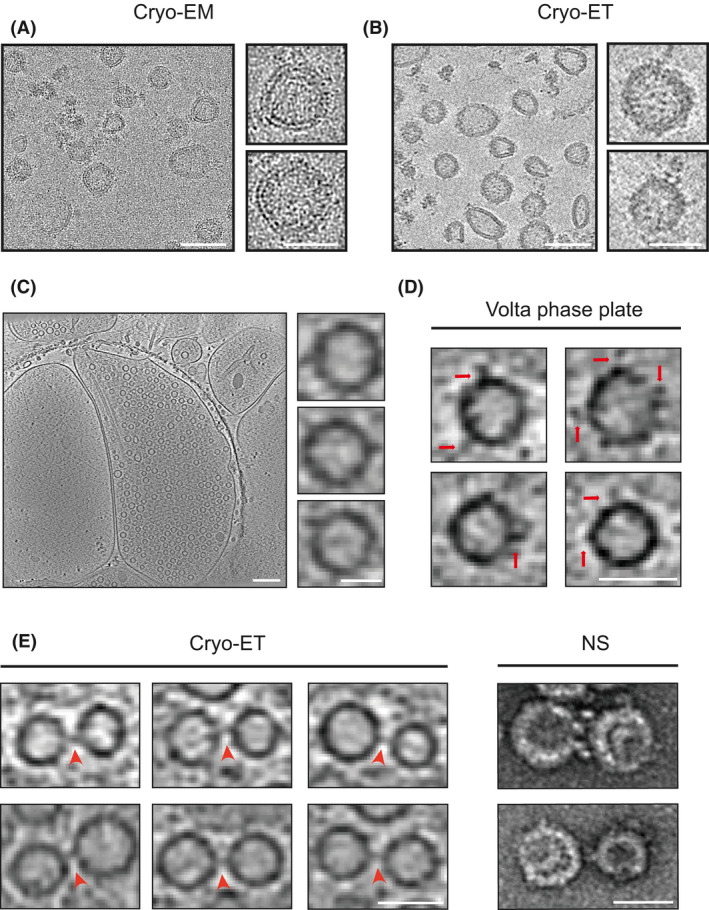
Visualization of SVs via cryo‐EM. (A) Cryo‐EM image of purified SVs. Scale bar = 100 nm. Zoomed scale bar = 50 nm. (B) a tomographic slice of purified SVs. Scale bar = 100 nm. Zoomed scale bar = 50 nm. (C) A tomographic slice showing primary cultured hippocampal neurons synaptic terminal. Scale bar = 200 nm. Zoomed scale bar = 50 nm. (D) Visualization of SVs in primary cultured hippocampal neurons applied VPP. Scale bar = 50 nm. (E) SV connectors (red arrows) in primary cultured hippocampal neurons based on cryo‐ET, and the purified SVs, negatively stained (NS) by uranyl acetate, also showed a similar connection. Scale bar = 50 nm.

Heavy‐metal staining is a rapid method for observing cellular ultrastructure, although fixation and dehydration of vesicles limit the understanding of molecular details [15, 16]. Recently, the development of cryo‐EM hardware and software has extended the range of observations of biological samples and the application for protein complexes has established high‐resolution structural processing [17]. Thus, we prepared a cryo‐EM sample of *in vitro* purified SVs from the mammalian brain. The vitrified, fully hydrated imaging provided an ellipsoidal and spherical shape of SVs (Fig. 3A). To further visualize morphological information of SVs, we applied cryo‐ET to our purified sample, and the generated tomograms showed a crowded distribution of membrane‐associated proteins to SVs (Fig. 3B). This observation is consistent with the recently reported proteomics of SVs [5], indicating that complicated protein interaction and regulation are required for diversified functions of SVs at presynaptic terminal. However, the heterogeneous distribution of coating proteins also limits effective identification of the copy number and type for these proteins, which may require hardware and structural algorithm optimization.

Next, we investigated whether morphological information of *in vitro* purified SVs represents a real state in cells. Mouse hippocampal neurons were isolated and cultured at 13–17 days *in vitro*, and thinner synapse regions allowed us to prepare eligible cryo‐ET samples (Fig. 3C). Consistent with the above results, vesicles at the presynaptic terminal also exhibited distinct coating proteins. Volta phase plate (VPP) has been reported to enhance contrast and signal‐to‐noise ratio of transmission electron microscopy records [18]; as expected, more structural details of SVs were provided with the utilization of VPP (Fig. 3D). Additionally, we also observed the interconnection of SVs at the active zone, consistent with previous reports [19, 20], and ‘short filaments’ of approximately 8–12 nm were clearly visualized between two vesicles (Fig. 3E). Surprisingly, in our *in vitro* purified SV, the connected SVs can also be found in well‐dispersed transmission electron microscopy images (Fig. 3E). Thus, although there are many successful protocols for the purification of SVs *in vitro*, the integrity of vesicles is required for subsequent biochemical characterization, and the rapid isolation method that we proposed here can effectively retain the comprehensive configuration of intact SVs.

## Discussion

Neurotransmitter release is crucial for neural information delivery. Neurotransmitters are released from the Ca^2+^ ions‐stimulated presynaptic terminal and captured by the postsynaptic side receptors to complete signal transmission [1, 2]. Synaptic vesicles are compartments stored with various neurotransmitters in the brain. Comprising the distal connection of neurons, synapses can be considered as a partially independent release system, which has high requirements for kinetics regulation of SVs. The exocytosis of SVs, including docking, priming and fusion, also requires precise control to respond to rapid stimuli. Various proteins/components and complicated interactions are the main participants in the above events.

Purification of SVs *in vitro* is essential for understanding intracellular processes, which allows for quantitative investigation by biochemical methods. In the past decades, many isolation protocols of vesicles have been established from cultured cells and mammalian brain, and detailed procedures have been modified to meet different needs [6, 7, 9]. However, multistep purifications reduce the yield and cause unpredictable damage to the vesicles. Here, we provide a simple method for isolating SVs from mouse brain, which can obtain qualified biological samples for biochemical characterization without density gradient centrifugation or column chromatography. Based on the observation of transmission electron microscopy, the isolated SVs retained intact shape and are close to the real situation in the synapse. Moreover, we also show that the isolated SVs were covered by rich proteins, indicating that SVs have unknown functions at the presynaptic terminal that need precise regulation. This observation was also verified by the cryo‐ET from neurons. Specifically, the filamentous connector between SVs has been proposed for the mobility and release of vesicles [19, 21, 22]. Of our isolated SVs, the connected vesicles were also reserved *in vitro* following rapid and gentle purification, indicating adequate strength of the connectors. Although the records from cryo‐EM provide abundant protein details, the heterogeneous profiles of vesicles‐associated proteins also bring limitations to high‐resolution structural assigned. Isolated SVs from our protocol will be suitable for comprehensive proteomic dissection, as well as for further analysis of proteins on SVs.

## Conflict of interest

The authors declare no conflict of interest.

## Author contributions

YX involved in conceptualization. LH, KD, PW, and YX involved in the methodology. LH, KD, PW, and YX involved in the investigation. LH and KD involved in writing—original draft. LH, KD, PW, and YX involved in writing—review and editing. YX involved in funding acquisition. YX involved in resources. YX involved in supervision.

## Data Availability

The data presented in this study are available from the corresponding author upon reasonable request.
